# Does sensorimotor upper limb therapy post stroke alter behavior and brain connectivity differently compared to motor therapy? Protocol of a phase II randomized controlled trial

**DOI:** 10.1186/s13063-018-2609-4

**Published:** 2018-04-20

**Authors:** Nele De Bruyn, Bea Essers, Liselot Thijs, Annick Van Gils, Lisa Tedesco Triccas, Sarah Meyer, Kaat Alaerts, Geert Verheyden

**Affiliations:** KU Leuven - University of Leuven, Department of Rehabilitation Sciences, Tervuursevest 101, box 1501, 3001 Leuven, Belgium

**Keywords:** Stroke, Upper limb, Sensorimotor function, Randomized controlled trial, Functional connectivity

## Abstract

**Background:**

The role of somatosensory feedback in motor performance has been warranted in the literature. Although sensorimotor deficits are common after stroke, current rehabilitation approaches primarily focus on restoring upper limb motor ability. Evidence for integrative sensorimotor rehabilitation approaches is scarce, as is knowledge about neural correlates of somatosensory impairments after stroke and the effect of rehabilitation on brain connectivity level. Therefore, we aim to investigate changes in sensorimotor function and brain connectivity following a sensorimotor therapy program compared to an attention-matched motor therapy program for the upper limb after stroke.

**Methods:**

An assessor-blinded randomized controlled trial will be conducted. Sixty inpatient rehabilitation patients up to eight weeks after stroke will be included. Patients will be randomized to either an experimental group receiving sensorimotor therapy or a control group receiving attention-matched motor therapy for the upper limb, with both groups receiving conventional therapy. Thus, all patients will receive extra therapy, a total of 16 1-h sessions over four weeks. Patients will be assessed at baseline, after four weeks of training, and after four weeks of follow-up. Primary outcome measure is the Action Research Arm Test. Secondary outcome measures will consist of somatosensory, motor and cognitive assessments, and a standardized resting-state functional magnetic resonance imaging protocol.

**Discussion:**

The integration of sensory and motor rehabilitation into one therapy model might provide the added value of this therapy to improve sensorimotor performance post stroke. Insight in the behavioral and brain connectivity changes post therapy will lead to a better understanding of working mechanisms of therapy and will provide new knowledge for patient-tailored therapy approaches.

**Trial registration:**

ClinicalTrials.gov, NCT03236376. Registered on 8 August 2017.

**Electronic supplementary material:**

The online version of this article (10.1186/s13063-018-2609-4) contains supplementary material, which is available to authorized users.

## Background

Patients surviving a stroke encounter a variety of impairments, one of which is a unilateral sensorimotor impairment in the upper limb. Somatosensory impairments are defined as deficiencies in the sensation arising from skin, muscles, or joints [1]. These impairments can consist of a deficit in discriminative abilities, proprioception, or detection of sensation such as light touch, pressure, or pain. Sensorimotor deficits at one week post stroke lead to limitations in activities of daily living and restrictions in participation in society [2]. In general, approximately half of the patients admitted to a stroke rehabilitation unit encounter somatosensory impairments [3, 4]. A recent study showed that at one week post stroke, 78% have a single or combined sensorimotor impairment in the affected upper limb [5].

Evidence-based guidelines for therapy aimed at restoring motor impairments do exist but, as yet, therapy-mediating somatosensory impairments are still scarce. A 2010 Cochrane review by Doyle et al. [6] on “interventions of sensory impairments in the upper limb after stroke” concluded that “*there is insufficient evidence to reach conclusions about the effects of interventions included in this review.”* In 2011, results from a randomized controlled trial (RCT) by Carey et al. [7] investigating the effect of upper limb sensory discrimination training on somatosensory function after stroke showed a significant effect of sensory discrimination training compared to general sensory exposure in chronic stroke [7]. Although the evidence for the effect of somatosensory therapy on somatosensory function is increasing, to our knowledge, only two case-series studies have investigated its effect on motor function [8, 9]. A preliminary study reported results of two patients who received sensorimotor stimulation training consisting of passive mobilization, sensory stimulation by means of an electric toothbrush for vibration, and position training for proprioception. The box and blocks test and the 10-s test were conducted after each session and showed improvements [9]. Another preliminary study reported a case series of two patients receiving a sensorimotor program consisting of manipulation tasks with a sensory discrimination aspect, such as sorting plastic eggs of different weights [8]. Also, the latter study reported improvement in motor function after training. Further, neural sensory reorganization was found on functional magnetic resonance imaging (fMRI) and brain volume measurements for both participants after the sensorimotor discrimination program [8].

The key role of somatosensory feedback in motor control and function has been investigated. Somatosensory impairments are associated with poorer dexterity, manipulation abilities, and bimanual hand coordination skills [6, 10, 11]. Impaired surface friction discrimination is associated with longer grip-lift latency times and increased grip force regulation during a grip-lift and hold task [12]. Intervention studies investigating the effect of somatosensory stimulation on motor learning in healthy controls confirmed the sensorimotor coupling. One study investigated the effect of repetitive transcranial magnetic stimulation over the primary somatosensory cortex, inhibiting somatosensory function, on motor learning [13]. They reported less accurate tracking and a smaller learning effect for a motor task during stimulation. These effects persisted at the retention test, indicating the influence of somatosensory disruption on motor control and learning [13–15]. Translating these paradigms to post-stroke therapy and designing an integrative treatment consisting of combined and emphasized somatosensory and motor input could provide additional sensorimotor benefit over exercises focusing on pure motor activation.

Besides behavioral outcomes, brain connectivity analysis post treatment would provide insights in neural correlates of upper limb therapy. Neural reorganization associated with stroke has also been mainly investigated for motor impairments. Increased intrahemispheric and decreased interhemispheric functional connectivity is associated with motor deficits [16–19]. Recovery of motor function is reported along normalization of inter- and intrahemispheric functional connectivity within the sensorimotor network of humans and rats [20–22]. However, knowledge about altered functional connectivity associated with somatosensory impairments is still limited. Results from our group showed decreased inter- and ipsilesional intrahemispheric functional connectivity in patients in the acute phase with severely impaired somatosensory function compared to patients with mild to moderate somatosensory impairments (unpublished data). Additionally, recovery of sensory discrimination is shown to be associated with an increase in interhemispheric functional connectivity between primary somatosensory cortices and both thalami [23]. To date, brain connectivity alterations in the sensorimotor network after sensorimotor vs motor therapy have not been investigated.

The role of resting state functional connectivity to serve as predictor for stroke recovery is still unclear. A recent position paper on “biomarkers of stroke recovery” [24] from the stroke recovery and rehabilitation roundtable suggests developmental priority to research concerning the utility of resting state functional connectivity to predict treatment response. Both for motor and somatosensory function, some but insufficient evidence is available to support the inclusion of resting state functional connectivity as a biomarker in clinical trials at present [24]. More specifically, the predictive value of interhemispheric resting state functional connectivity at baseline can be investigated to determine its potential to serve as biomarker. The future inclusion of biomarkers as eligibility criterion in clinical trials will result in more homogeneity within the study population and a better estimation of treatment effect. Therefore, including brain imaging outcome parameters in clinical trials will provide insights in underlying mechanisms of therapy-induced effects at brain level as well as suggesting biomarkers to be considered for future clinical trials.

To summarize, somatosensory function plays an important role in motor learning and performance. Sensory discrimination therapy has a positive effect on recovery of somatosensory function post stroke but the effect on motor behavior and underlying brain connectivity alteration is not fully understood. Therefore, we will conduct a phase II RCT with two parallel groups to investigate the effect of sensorimotor vs motor therapy on sensorimotor function and brain connectivity. We hypothesize that after sensorimotor therapy, patients will show a greater improvement in sensorimotor function compared to patients receiving motor therapy. We further hypothesize that brain connectivity changes related to sensorimotor recovery will show increased normalization within the sensorimotor network compared to patients receiving motor therapy.

## Methods

### Study design

**Fig. 1:**
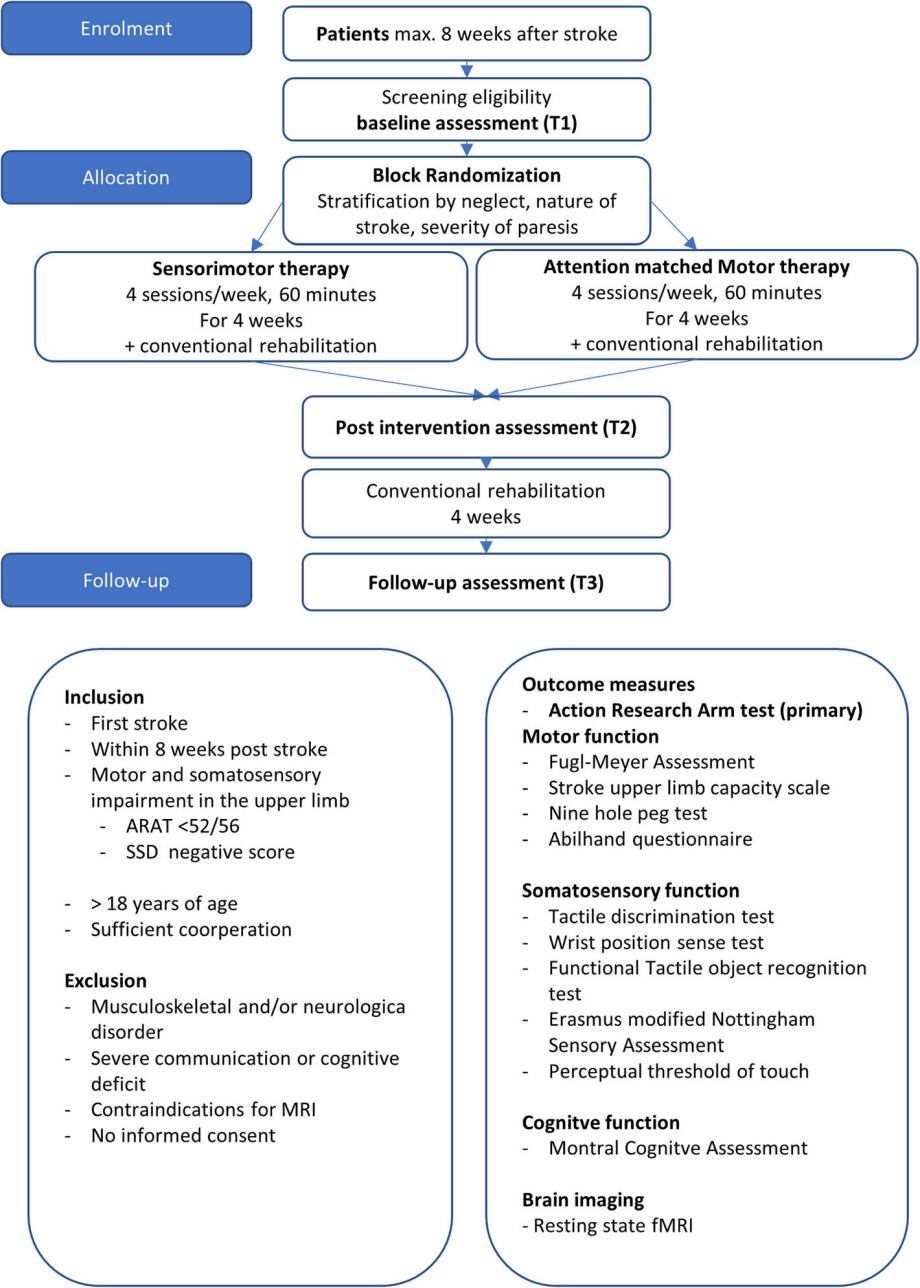
Study *flow diagram*

This study is an assessor-blinded multicenter RCT. Integrative and emphasized sensorimotor upper limb therapy will be compared to attention-matched motor upper limb therapy. Both groups will receive the same amount of therapy contact and training time as part of this therapy, in addition to conventional patient-focused inpatient rehabilitation. An overview of the study flow diagram is provided in Fig. 1. Unblinding of the assessor will not be permissible at any time point.

The study is reported conform the SPIRIT (Standard Protocol Items: Recommendations for Interventional Trails) statement [24] (Additional file 1).

### Study setting

This multicenter RCT will be conducted by the University of Leuven (KU Leuven) at the stroke ward of two rehabilitation centers in Flanders, Belgium: University Hospitals Leuven (Campus Pellenberg) and Jessa Hospital (Campus Sint-Ursula, Herk-de-Stad).

### Participants

Sixty patients after first-ever unilateral, supra-tentorial stroke as defined by the World Health Organization [25] will be included after admission to the rehabilitation center with following inclusion criteria: (1) stroke onset within eight weeks; (2) presence of motor and somatosensory impairment in the upper limbs; (3) aged 18 years and older; and (4) providing substantial cooperation. Motor impairment is defined as a score < 52/57 on the Action Research Arm Test (ARAT). Somatosensory impairment is defined as a negative score on the standardized somatosensory deficit composite score [7].

Patients with following criteria will be excluded: (1) musculoskeletal or other neurological disorders with stroke-like symptoms; (2) severe communication or cognitive deficits; (3) the presence of contra indications for undergoing an MRI brain imaging protocol; and (4) no informed consent.

### Randomization

Based on a computer-generated list, a stratified block randomization has been performed with an allocation ratio of 1:1 for both therapy groups (integrative and emphasized sensorimotor upper limb therapy and attention-matched motor upper limb therapy). We stratify our included patients for neglect (cut-off score < 44 out of 54 on the star cancellation task [26, 27]), for ischemic or hemorrhagic stroke, and for mild to moderate paresis (minimum 10° active wrist and finger extension) or severe paresis (no active wrist and finger extension). Based on information concerning the stratification factors, an independent researcher (GV) provides concealed allocation through informing the therapist (ND) of the random allocation. Also, the assessor remains blinded for group allocation as this is an independent, third researcher.

### Procedure

**Fig. 2:**
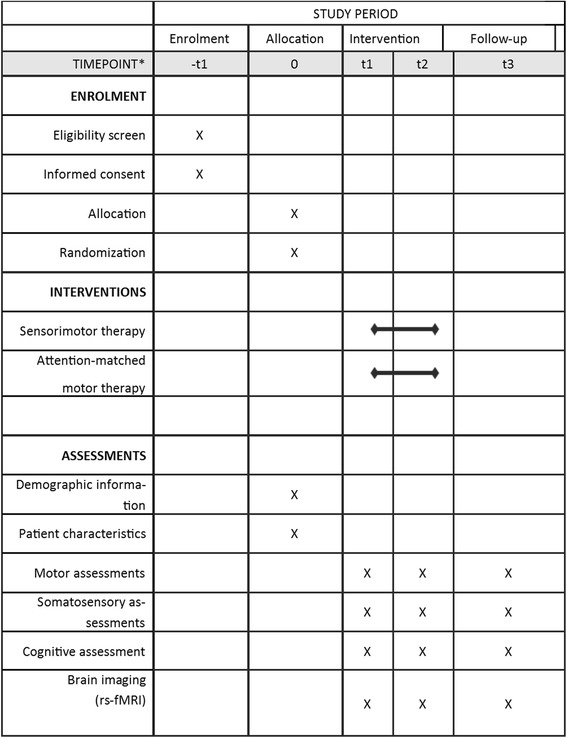
Schedule of enrolment, interventions, and assessment following SPIRIT guidelines. T1 baseline assessment, T2 assessment after four weeks of intervention, T3 assessment after four-week follow-up (eight weeks after start of intervention)

An overview of the study flow diagram is provided in Figs. 1 and 2.

#### Screening and consent

After admission to the centers, patients will be screened on eligibility based on experience of the (para)medical staff members. A visit will be planned for eligible patients by the therapist (ND) to inform them about the content and aims of the study and to hand over the information folder. After providing informed consent to the therapist (ND), the blinded assessor (LT) will be informed by the therapist that the patient is enrolled in the trial. A third researcher (GV) receives the necessary information and allocates the included patient to one of the treatment groups based on the computer-generated list.

#### Intervention

Patients will receive standard conventional rehabilitation as well as an additional four-week intervention. The additional intervention comprises 16 h; four 1-h training sessions per week for four weeks by the therapist (ND) in a one-to-one setting. Content of the delivered additional therapy will be recorded on paper (all sessions) and video (one random session for each patient). The amount of conventional rehabilitation, such as physical and occupational therapy actually received, will also be registered for each patient.

##### Sensorimotor therapy

The sensorimotor therapy will consist of 30 min of somatosensory discrimination training and 30 min of integrative and emphasized sensorimotor training per session in a one-to-one setting. The sensory discrimination training will be based on the SENSe approach [7] and will include three sensory discrimination tasks: texture discrimination; limb position sense; and tactile object recognition. Texture discrimination will be trained using progressively and variated surface characteristics such as fabric, wallpaper, plastic, and sandpaper. Limb position sense will be trained across a wide range of upper limb positions and include for instance full flexion, full extension, and neutral position of the index finger, wrist, and elbow. Tactile object recognition will be trained using different objects varying of shape, size, weight, texture, hardness, and temperature such as bottles with differences in amount of contained fluid, cutlery, plates of different materials such as wood, plastic, metal, coins of different sizes, etc. An overview of examples of possible exercises can be found in Additional file 2. The 30-min sensorimotor training per session will be the same individually tailored motor therapy as described below, but with full integration of sensory discrimination training aspects such as reaching towards and bringing to the mouth the same bottles with varying weights used in the sensory part of the therapy with the additional task to determine the weight of the bottle. A well-defined package of exercises has been developed in order to standardize this therapy approach.

##### Motor therapy

The motor therapy will consist of 30 min of cognitive and attention-based table top games and 30 min of motor training per session in a one-to-one setting. The cognitive attention-based therapy will consist of table top games such as chess, rush hour, or other smart games, all performed with the unaffected upper limb. Individually tailored motor therapy consists of a unilateral motor exercise program for the affected upper limb, while seated at a table, under supervision of a therapist to match the therapy and intensity provided in the sensorimotor therapy group. This 30-min motor arm training will be based on a set of standardized exercises which comprise of task-related practice for gross movements and dexterity including different grips and selective finger movements, and training in daily life activities, however without any attention to sensory discrimination training [28]. A complete package of exercises has been developed for a recently conducted trial [28] and this will also be applied in this trial.

#### Safety

We collect adverse events from our participants through asking our patients before the next treatment or assessment session whether any event occurred since the previous session. Events formulated will be evaluated for causality and if linked to the treatment provided, this will be reported as an adverse reaction or serious adverse reaction (in case of a life-threatening complication, prolonged hospitalization, or persistent and significant remaining disability) although the latter is unlikely based on the type of treatment provided in this trial.

### Outcome measures

All patients will be assessed with the protocol described in detail below by a trained assessor (LT) who is blinded for group allocation. Patients will be assessed before the intervention (pre-intervention; T1), immediately after the intervention (post-intervention; T2) and four weeks after the end of the intervention (follow-up; T3). At baseline (T1: pre-intervention), demographic data and baseline patient characteristics such as age, gender, time post stroke, lateralization of symptoms, and hand dominance as assessed by the handedness questionnaire [29] will be recorded. Cognitive impairment will be assessed by the Montreal Cognitive Assessment [30], tactile extinction by the perceptual threshold of touch [31], and visuo-spatial neglect by the star cancellation test [26]. At baseline (T1), post-intervention (T2), and follow-up (T3) a standardized sensorimotor examination combined with resting-state fMRI brain imaging protocol will be performed (Table 1).

**Table 1:** Outcome measures

Primary	ARAT	Action Research Arm Test [32]: grasp, grip, pinch, and gross movement of the affected upper limb
Secondary	FMA-UE	Fugl-Meyer Motor Assessment - Upper Extremity [34]: overall motor impairment of the affected upper limb: shoulder, arm, wrist, hand, and fingers
	9HPT	Nine-Hole Peg Test [36]: manual dexterity
	SULCS	Stroke Upper Limb Capacity Scale [37]: upper limb capacity
	ABIL	Abilhand questionnaire [38]: perceived hand function
	Em-NSA	Erasmus modified Nottingham Sensory Assessment [39]: light touch, pressure, sharp, sharp-dull discrimination, position sense of the upper limb
	PTT	Perceptual threshold of touch [31]: light touch
	SSD	Composite standardized somatosensory deficit index [7]:
	TDT	Texture discrimination test [41]: texture discrimination
	WPST	Wrist position sense test [42]: position sense
	fTORT	Functional Tactile Object Recognition Test [43]: recognition of object by touch
	MoCa	Montreal Cognitive Assessment [30]: cognitive function
Brain imaging protocol		
Resting-state functional magnetic resonance imaging (rs fMRI)		
	Anatomical MRI scan Resting state fMRI scan	
		Standardized 3 Tesla MRI scanner, including standard head coil 30 parallel transverse orientated slices (thickness 4 mm, no interslice gap) Parameter settings: TE = 33 ms, flip angle = 90°, TR = 1700 ms, FOV = 230 mm, matrix = 64 × 64 Duration: 7 min

*TE* echo time, *TR* repetition time, *FOV* field of view

Our primary outcome measure is the ARAT. The ARAT consists of four subscales: grasp; grip; pinch; and gross movement to evaluate the activity level of the upper limb. A total score between 0 (loss of motor performance) and 57 (good motor performance) can be obtained. Adequate reliability and validity are reported in the literature [32]. We hypothesize that combined and emphasized sensorimotor therapy will benefit motor function more than pure motor therapy due to sensorimotor coupling. Therefore, we have chosen the ARAT as measurement of upper limb function, which is widely used in research as primary outcome measure and a recommended measurement tool for upper limb activity [33].

Secondary motor outcome measures are the Fugl-Meyer assessment - upper extremity part (FMA-UE) [34] at function level according to the ICF model [35], the Nine-Hole Peg Test (9HPT) [36] evaluating dexterity, the Stroke Upper Limb Capacity Scale (SULCS) [37] investigating basic and extended upper limb activity, and the Abilhand questionnaire (ABIL) for perceived manual ability [38].

Secondary somatosensory outcome measures include the Erasmus-modified Nottingham Sensory Assessment (Em-NSA) [39] and perceptual threshold of touch (PTT) [31]. Additionally, the composite standardized somatosensory deficit score [40] consisting of the tactile discrimination test (TDT) [41], wrist position sense test (WPST) [42], and functional tactile object recognition test (fTORT) [43] will be performed to investigate somatosensory function.

All tests have good psychometric properties. Additional file 3 provides more information about content and psychometric properties of the outcome measures. Finally, brain imaging is conducted and will consist of a standardized protocol including an anatomical scan and a resting-state fMRI scan. A total of 30 parallel slices of 4-mm thickness and transverse orientated with no interslice gap will be obtained for the resting state fMRI scan. Scanning parameters include echo time (TE) = 33 ms, repetition time (TR) = 1700 ms, flip angle = 90°, field of view (FOV) = 230 mm, matrix = 64 × 64, and duration = 7 min. Patients will be in the supine position with eyes open and fixating on a cross and will be asked to not think about anything in particular or fall asleep.

### Sample size and power

This trial is considered an “effect and mechanism” study investigating the effect of intervention in relation to mechanisms of recovery and therefore including the combination of clinical and brain imaging methodology. We aim to recruit 60 participants in a two-year period – 30 in each group. For the present phase II trial, the sample size was based on comparable studies.

At the end of our trial, a sample size calculation for a definitive phase III trial will be conducted.

### Data analysis

In order to evaluate if randomization was successful for baseline characteristics, groups will be compared at pre-intervention. For our primary and secondary outcome measures, between-group intention-to-treat analysis will consist of comparing differences between both groups of pre vs post and pre vs follow-up scores for the motor and somatosensory measures based on mixed model analysis. Sub-group analyses will be conducted for patient groups according to neglect, severity of upper limb paresis, and level of cognition. We will further evaluate the between-group difference in proportion of responders vs non-responders for motor and somatosensory function, based on the minimal detectable change for our outcome measures. Subsequently, characteristics of responders and non-responders will be investigated by means of uni- and multivariate regression analysis.

Brain imaging analysis will consist of pre-processing and region of interest (ROI)-to-ROI analysis of the resting-state fMRI data. A full factorial design will be obtained to investigate the group and interaction effects between stroke and a group of age- and gender-matched healthy participants for both clinical differential scores and for resting-state fMRI values. General linear models and partial correlations will be performed to investigate the brain-behavior relationships between both trial groups, with center, mean frame wise displacement, age, and neglect as covariates of no interest. False discovery rate correction will be implemented as correction for multiple comparison.

#### Patient drop-out

When patients drop out at T2, they will be contacted to participate at the follow-up measurement (T3), when possible.

#### Data management

Data from clinical assessments will be recorded in paper booklets for each patient and converted with double data entry into a PC data file stored on a secured server of the university of Leuven. Brain imaging data will be saved on an external drive to transport data towards the same secured drive. Participants’ paper files will be kept for storage for a period of 20 years and electronic data will be stored on a secured server. Confidential information will be converted into unique codes and the key to these codes will be kept in a separate file on a password-protected electronic location.

### Ethical considerations

For this study, we obtained approval from both the ethical committees of KU Leuven/University Hospitals Leuven and Jessa Hospital and this trial complies to the principles of the “declaration of Helsinki.” Before the start of participation in this trial, written informed consent will be obtained. This trial is registered at ClinicalTrials.gov (NCT 03236376). All data collected during this study will be confidential but with open access for key staff members. To protect the privacy of the participants, they will be given unique numerical codes. The key to this code will be maintained securely and confidentially by ND. No information with which the participant can be identified will be available in any other study-related (electronic) document. All assessment forms, reports, and other (electronic) records will be coded and handled to maintain strict participant confidentiality.

### Modification of the protocol

Any important modification of the protocol concerning assessments, participant inclusion, or administrative aspects will be communicated by a formal amendment. Such amendment will need to be approved by the ethical committee of KU Leuven/University Hospitals Leuven before implementation and communication to the relevant parties.

## Discussion

This study aims to investigate the effect of integrative and emphasized sensorimotor therapy compared to attention-matched motor therapy in addition to conventional therapy, on behavioral and brain parameters. We hypothesize that sensorimotor function will improve more in patients who received the sensorimotor therapy compared to patients of the motor therapy group. Additionally, we hypothesize that brain connectivity changes are related to recovery of sensorimotor function, with more increased normalization within the sensorimotor network for the sensorimotor therapy group.

The strengths of this study are the integrative approach of the sensorimotor therapy program and the combined clinical assessment and brain imaging analysis of our participants. In order to find optimal rehabilitation paradigms, knowledge about optimal timing and spontaneous and rehabilitation-induced plasticity of the brain is needed. Furthermore, the use of biomarkers to predict responders and non-responders will help to provide more patient-tailored therapy in the future. To get more insights in biomarkers of stroke recovery and underlying mechanisms of therapy-induced effects, an integrative approach combining clinical and brain imaging outcome measurements is suggested. Therefore, we will examine the effects of our sensorimotor therapy model both on clinical as well as brain imaging outcomes. This will lead to better understanding of the neural mechanisms underlying behavioral improvement.

Second, with this RCT, we investigate the effect of a novel sensorimotor program on upper limb function. To our knowledge, this kind of therapy is under-investigated. Somatosensory function is reported to be associated with motor and functional outcome after stroke [4–6, 12] and somatosensory impairment is reported to have a negative influence on motor recovery [10]. To date, only two studies investigating a sensorimotor program on motor function are reported [8, 9]. Both studies were case series but did report improved scores on motor function after training. Our trial with an RCT design will provide additional knowledge concerning clinical effect of sensorimotor training on motor as well as somatosensory function.

Despite the strengths of our study, some challenges remain. Good clinical assessments for motor and somatosensory function often lack sensitivity to change. We composed an extensive and established assessment battery of tests covering both function and activity level. Both assessments of upper limb motor (FMA) and somatosensory (Em-NSA) function as well as more specific assessments for dexterity (9HPT), proprioceptive (WPST), or touch discriminative function (TDT) will be included in the protocol to cover a wide spectrum of somatosensory and motor function, and activity level performance of patients [34, 36, 39, 41, 42]. Additionally, the inclusion of the perceptual threshold of touch as an outcome measure for light touch will address the sensitivity since it is a more objective and a ratio-scaled outcome measure [31]. For patients with severe communication difficulties, assessment of somatosensory function may be challenging. In addition, attention deficits could influence somatosensory assessment. When somatosensory function is decreased and patients do not pay attention to the somatosensory stimuli during assessment, they could incorrectly report not to feel the stimulus. Therefore, patients with severe communication and attention disabilities are excluded from our trial, which will limit the generalizability of our findings.

In summary, this trial will investigate the behavioral and neural correlates associated with stroke-induced sensorimotor therapy for the upper limb. More specifically, the effects of therapy on clinical outcome and functional connectivity in rest is the scope of this study. Thus, our findings will provide novel insights in the neural base of recovery of upper limb sensorimotor function after stroke.

## Trial status

Recruitment started on 1 October 2017 and will end on 30 November 2019 or when 60 participants are recruited to the trial.

## Additional files

Additional file 1: SPIRIT checklist; checklist based on the SPIRIT guidelines. (DOCX 19 kb)

Additional file 2: Examples of sensorimotor training tasks, overview of examples of exercises performed by the patients allocated in the sensorimotor trainings group. (DOCX 13 kb)

Additional file 3: Outcome measures. (DOCX 26 kb)

## Abbreviations

9HPT: Nine-Hole Peg Test
ABIL: Abilhand questionnaire
ARAT: Action Research Arm Test
Em-NSA: Erasmus modified Nottingham Sensory Assessment
FMA-UE: Fugl-Meyer Motor Assessment - Upper Extremity
fMRI: Functional magnetic resonance imaging
FOV: Field of view
fTORT: Functional Tactile Object Recognition Test
ICF: International Classification of Functioning, Disability and Health
n: Number
PTT: Perceptual threshold of touch
RCT: Randomized controlled trial
ROI: Region of interest
rs fMRI: Resting state functional magnetic resonance imaging
SPIRIT: Standard Protocol Items: Recommendations for Interventional trials
SSD: Composite standardized somatosensory deficit index
SULCS: Stroke Upper Limb Capacity Scale
T1: Assessment at pre-intervention
T2: Assessment at post-intervention
T3: Assessment at follow-up
TDT: Texture Discrimination Test
TE: Echo time
TR: Repetition time
WHO: World Health Organization
WPST: Wrist position sense test
