# Supplementary material for: Does sensorimotor upper limb therapy post stroke alter behavior and brain connectivity differently compared to motor therapy? Protocol of a phase II randomized controlled trial
Source: Trials. 2018 Apr 20;19:242. doi: 10.1186/s13063-018-2609-4 (PMC5910616; doi:10.1186/s13063-018-2609-4)
Supplement: Supplementary file 2 — Examples of sensorimotor training tasks, overview of examples of exercises performed by the patients allocated in the sensorimotor trainings group. (DOCX 13 kb) [file 13063_2018_2609_MOESM2_ESM.docx]

| **Training modality** | **Task** | **Description** |
| --- | --- | --- |
| Texture discrimination | Forward sliding over table with different surfaces | Slide with your hand over the surfaces (lego paper model, smooth plastic placemat, handicraft foamsheet), which are placed on the table. Surfaces are changed between sliding movements and patients are asked to feel the differences in surface. |
|  | Smooth out a crumbled fabrics | Smooth out consecutively a crumbled dishtowel, a bath towel and a cotton t-shirt with your affected hand and try to feel the differences in structure while smoothing. |
| Limb position sense | Mirror movement with the affected limb | Try to position the joint (finger, wrist or elbow) of you affected limb into the same position as the therapist has positioned your unaffected limb with your eyes closed. |
|  | Movement exercise with visual feedback | Try to move your joint from one point to the other. A pen will be attached to the moving joint to draw the movement pattern of the patient. After a few times of exercising the movement with eyes open, you try the same movement with eyes closed. Feedback is given based on the drawing pattern. |
| Tactile object recognition | Sort bottles of different weight | Sort the bottles by weight. Full range of bottles consist of pairs of empty, ¼ filled, ½ filled, ¾ filled and fully filled bottles. The number of bottles and difference in weight depends on the level of sensory discrimination obtained during the first 30 minutes of SENSe training. |
|  | Selecting cutlery | Identify a specific piece of cutlery (knife, fork, spoon, and teaspoon) in the bag and take it out of the bag. |
|  | Selecting coins | Coins of different sizes are collected in a wallet. Take a specific amount of money out of the wallet and put it in to the piggy bank with vision occluded from the inside of the wallet. |
|  | Open and close clothing | Open and close buttons, zippers, Velcro and push buttons with and without eyes closed. |
|  | Bringing cups to the mouth | Bring cups with different crushability (glass, paper, disposable cups, plastic cups) to the mouth and back to the table |
|  | Sorting ice cubes | Sort frozen and room temperature plastic ice cubes |
|  | Sorting cards | Sort cards with different textures (paper card, business card, laminated card) |
