# Supplementary material for: Does sensorimotor upper limb therapy post stroke alter behavior and brain connectivity differently compared to motor therapy? Protocol of a phase II randomized controlled trial
Source: Trials. 2018 Apr 20;19:242. doi: 10.1186/s13063-018-2609-4 (PMC5910616; doi:10.1186/s13063-018-2609-4)
Supplement: Supplementary file 3 — Outcome measures. (DOCX 26 kb) [file 13063_2018_2609_MOESM3_ESM.docx]

The FMA-UE examines overall motor impairment of the affected upper limb. Scores range between 0 (loss of motor function) and 66 (intact motor function). The FMA has good psychometric properties [1]. The 9HPT examines manual dexterity [2]. Age-, gender- and hand dominance specific norm values for healthy subjects are available [2], and comparative values for one, three and six months after stroke are reported in literature [2]. Adequate test-retest, intra- and interrater reliability and validity have been reported [2]. The SULCS [3] is a recent-developed unidimensional, hierarchical clinical assessment tool to evaluate upper limb activity by means of ten functional and meaningful tasks related to daily live activities, such as taking support on the affected limb, bringing a glass to the mouth, and closing buttons. A total score between zero (severe motor limitations) and ten (mild to no motor limitations) can be obtained. The SULCS has good interrater reliability and construct validity [4]. The Abilhand questionnaire is a questionnaire to measure perceived difficulties with tasks of manual ability. Rash-based validation and excellent reliability have been reported [5].

The Em-NSA examines the detection of light touch (cotton wool), pressure (index finger) and pinprick (toothpick) at predefined contact points on the affected upper limb. Additionally, sharp-dull discrimination is evaluated with random alternating pressure (index finger) and pinprick (toothpick) stimulation at the same predefined contact points. Finally, movement sense (proprioception) will be evaluated at predefined joints of the upper limb. Scores ranges between 0 and 8 for each modality, with higher scores indicating better somatosensory function [6]. The Em-NSA is reported to be a reliable and valid assessment tool for somatosensory function [6, 7]. The PTT evaluates the threshold of light touch of the fingertip of the index finger using TENS (transcutaneous electric nerve stimulation) with the portable device CEFAR Primo Pro (Cefar Medical AB, Sweden). Round electrodes are attached to the tip of the index finger and the ball of the thumb. Single square pulses of 80µs pulse duration of 40Hz high frequency constant current are applied gradually increasing the amplitude with increments of 0.5 mA until the moment the patient reports a tingling sensation at the fingertip. Due to safety regulations, a maximum of 10 mA is used [8]. If patients do not report any sensation at 10mA a score of 11 is given. Adequate reliability has been reported [8]. Gender- and age-dependent norm values are available [9].

Furthermore, TDT evaluates texture discrimination by presenting different texture sets consisting of three textures, two identical and one different. The patient is asked (with vision occluded) to discriminate between the textures and to indicate which of texture is different compared to the other two textures. The TDT has a good test-retest reliability [10]. The WPST evaluates position sense (proprioception). The arm and hand of the patient will be positioned in two splints assuring fixed position of the arm and hand. The assessor will position the wrist in twenty predefined positions at random sequence and with vision occluded. A scale is provided for the patient above the wrist. The patient is asked to match the position of the lever on the scale with the position of the wrist. Degrees of error between the estimated position and the actual position will be noted. A total and mean error score will be calculated. Reliability has been reported [11]. The fTORT evaluates the ability to identify objects by means of touch and manipulation (stereognosis) [12]. A poster with every day common objects and distractor objects will be provided to the patient. The patient will be asked to indicate the photograph that corresponds to the object he/she will be feeling within 120 seconds (vision occluded). In case of paresis negatively influencing the ability to manipulate, the assessor may provide assistance to manipulate the object. Total score ranges between 0 (no object correctly identified) to 42 (all 14 objects correctly identified). Additionally, time needed to identify the object and the exploratory procedures, based on haptic perception [13], are recorded for each object. Age-adjusted normative standards, high reliability and good discriminative test properties are reported [12]. The composite standardized somatosensory deficit score will be composed by the average of the three z-transformed scores for TDT, WPST and fTORT.

**References**

1. Fugl-Meyer AR, Jaasko L, Leyman I, Olsson S, Steglind S. The post-stroke hemiplegic patient. 1. a method for evaluation of physical performance. Scandinavian journal of rehabilitation medicine. 1975; 7(1):13-31

2. Mathiowetz V, Weber K, Kashman N, Volland G. Adult norms for the nine hole peg test of finger dexterity. The Occupational Therapy Journal of Research. 1985; 5(1):24-38

3. Roorda LD, Houwink A, Smits W, Molenaar IW, Geurts AC. Measuring upper limb capacity in poststroke patients: development, fit of the monotone homogeneity model, unidimensionality, fit of the double monotonicity model, differential item functioning, internal consistency, and feasibility of the stroke upper limb capacity scale, SULCS. Archives of physical medicine and rehabilitation. 2011; 92(2):214-27.10.1016/j.apmr.2010.10.034.

4. Houwink A, Roorda LD, Smits W, Molenaar IW, Geurts AC. Measuring upper limb capacity in patients after stroke: reliability and validity of the stroke upper limb capacity scale. Archives of physical medicine and rehabilitation. 2011; 92(9):1418-22.10.1016/j.apmr.2011.03.028.

5. Penta M, Tesio L, Arnould C, Zancan A, Thonnard JL. The ABILHAND questionnaire as a measure of manual ability in chronic stroke patients: Rasch-based validation and relationship to upper limb impairment. Stroke. 2001; 32(7):1627-34

6. Stolk-Hornsveld F, Crow JL, Hendriks EP, van der Baan R, Harmeling-van der Wel BC. The Erasmus MC modifications to the (revised) Nottingham Sensory Assessment: a reliable somatosensory assessment measure for patients with intracranial disorders. Clinical rehabilitation. 2006; 20(2):160-72

7. Wu CY, Chuang IC, Ma HI, Lin KC, Chen CL. Validity and Responsiveness of the Revised Nottingham Sensation Assessment for Outcome Evaluation in Stroke Rehabilitation. The American journal of occupational therapy : official publication of the American Occupational Therapy Association. 2016; 70(2):7002290040p1-8.10.5014/ajot.2016.018390.

8. Eek E, Engardt M. Assessment of the perceptual threshold of touch (PTT) with high-frequency transcutaneous electric nerve stimulation (Hf/TENS) in elderly patients with stroke: a reliability study. Clinical rehabilitation. 2003; 17(8):825-34

9. Eek E, Holmqvist LW, Sommerfeld DK. Adult norms of the perceptual threshold of touch (PTT) in the hands and feet in relation to age, gender, and right and left side using transcutaneous electrical nerve stimulation. Physiotherapy theory and practice. 2012; 28(5):373-83.10.3109/09593985.2011.629021.

10. Carey LM, Oke LE, Matyas TA. Impaired touch discrimination after stroke: a quantiative test. J Neurol Rehabil. 1997; 11(4):219-32

11. Carey LM, Oke LE, Matyas TA. Impaired limb position sense after stroke: a quantitative test for clinical use. Archives of physical medicine and rehabilitation. 1996; 77(12):1271-8

12. Carey LM NJ, LeBlanc S, Harvey L. . A new functional tactual object recognition test (fTORT) for stroke clients: normative standards and discriminative validity. Paper presented at: 14th International Congress of the World Federation of Occupational Therapists; Sydney, Australia. 2006;

13. Lederman SJ, Klatzky RL. Hand movements: a window into haptic object recognition. Cogn Psychol. 1987; 19(3):342-68
